# Scanning Probe Microscopies: Imaging and Biomechanics in Reproductive Medicine Research

**DOI:** 10.3390/ijms22083823

**Published:** 2021-04-07

**Authors:** Laura Andolfi, Alice Battistella, Michele Zanetti, Marco Lazzarino, Lorella Pascolo, Federico Romano, Giuseppe Ricci

**Affiliations:** 1 Istituto Officina dei Materiali IOM-CNR, 34149 Trieste, Italy; battistella@iom.cnr.it (A.B.); zanetti@iom.cnr.it (M.Z.); lazzarino@iom.cnr.it (M.L.); 2 Doctoral School in Nanotechnology, University of Trieste, 34100 Trieste, Italy; 3 Institute for Maternal and Child Health, IRCCS Burlo Garofolo, 34137 Trieste, Italy; lorella.pascolo@burlo.trieste.it (L.P.); federico.romano@burlo.trieste.it (F.R.); 4 Department of Medical, Surgical and Health Sciences, University of Trieste, 34127 Trieste, Italy

**Keywords:** AFM, SNOM, spermatozoa, ovary, oocyte, IVF, embryos, blastocysts

## Abstract

Basic and translational research in reproductive medicine can provide new insights with the application of scanning probe microscopies, such as atomic force microscopy (AFM) and scanning near-field optical microscopy (SNOM). These microscopies, which provide images with spatial resolution well beyond the optical resolution limit, enable users to achieve detailed descriptions of cell topography, inner cellular structure organization, and arrangements of single or cluster membrane proteins. A peculiar characteristic of AFM operating in force spectroscopy mode is its inherent ability to measure the interaction forces between single proteins or cells, and to quantify the mechanical properties (i.e., elasticity, viscoelasticity, and viscosity) of cells and tissues. The knowledge of the cell ultrastructure, the macromolecule organization, the protein dynamics, the investigation of biological interaction forces, and the quantification of biomechanical features can be essential clues for identifying the molecular mechanisms that govern responses in living cells. This review highlights the main findings achieved by the use of AFM and SNOM in assisted reproductive research, such as the description of gamete morphology; the quantification of mechanical properties of gametes; the role of forces in embryo development; the significance of investigating single-molecule interaction forces; the characterization of disorders of the reproductive system; and the visualization of molecular organization. New perspectives of analysis opened up by applying these techniques and the translational impacts on reproductive medicine are discussed.

## 1. Multifunctional and High-Resolution Imaging Techniques for Application in Reproductive Medicine

Basic research in the reproductive medicine field has led to the development of the assisted reproductive techniques (ARTs) that can overcome human infertility. Like other biology areas, this research field benefits from implementing new technological approaches [1] and advanced ultrasensitive spectroscopic techniques [2,3,4,5] to deepen the knowledge of the properties and functions of single proteins, cells, or tissues. The aim is to improve the diagnosis and management of reproductive disorders. It would be enormously beneficial to adopt a multidisciplinary approach involving medical doctors, biologists, and physicists. In particular, the application of biophysical tools may disclose new features of gametes, cells, and tissues of the reproductive system that could help bridge the gap in the understanding of many reproductive pathophysiology aspects. 

The visualization of cell ultrastructure, macromolecular organization, and protein dynamics in living cells provides relevant insights to identify and then clarify the molecular mechanisms involved in biological processes of the reproductive system. 

Several imaging techniques based on material optical properties (e.g., birefringence) or on fluorescent dyes (e.g., epifluorescence, confocal, and light-sheet microscopy) have been exploited to characterize the structural organization of gametes and embryos. Essential contributions to the knowledge of these cells were also provided by nonlinear optical approaches [6,7]. Other label-free techniques based on vibrational signals, such as Raman microspectroscopy and Fourier transform infrared spectroscopy, represent valuable complementary techniques to obtain information about the biochemical compositions of molecular elements (DNA damage, lipid component, etc.) of gametes and embryos [2,5,6,8], with relevant contributions also in the studies of reproductive organs [9].

While the resolution offered by typical optical microscopies is sufficient to study, e.g., whole-cell or tissue morphology, many subcellular structures and processes remain elusive, hindered by the diffraction limit. For this reason, the biophysical community has directed enormous efforts to developing new super-resolution optical and fluorescence approaches for live imaging, such as total internal reflection fluorescence (TIRF) and stimulated emission depletion (STED), which improved the study of localization and dynamics of biomolecules and organelles in cells at the nanoscale level [10], as in embryo drosophila development [11]. This family of highly sensitive microscopies also includes scanning near field optical microscopy (SNOM), which combines super-resolution optical microscopy with a topographical image. A better topographical resolution can be obtained by atomic force microscopy (AFM). AFM topographic images have been demonstrated to be a powerful complement to fluorescence and electron microscopies, offering the possibility to visualize and investigate living cells with high resolution and in near-physiological conditions.

Moreover, to improve the knowledge of the molecular mechanisms involved in the reproductive system, it is crucial to analyze other biological properties and processes. The emerging field of mechanobiology, at the interface of biology, physics, and engineering, has highlighted the importance of mechanics in biological processes [12]. With a process called mechanotransduction, the cell transduces a physical stimulus into a signal cascade by which different molecular mechanisms are triggered, such as gene replication, gene transcription, and protein expression. The importance of mechanotransduction events in tissue morphogenesis, developmental processes, cell differentiation, and disease development is becoming widely accepted [13,14]. It is now well known that the mechanical status of cells and tissues is related to normal or altered molecular mechanisms. To describe this relationship, it is essential to develop techniques able to apply and measure very gentle and localized forces comparable, in intensity and point of application precision, with the molecular ones. Progresses in this field are supported by the development of advanced tools such as optical tweezers, particle-tracking microrheology, traction force microscopy, and AFM [15]. AFM provides a versatile platform for mechanobiology studies and enables the investigation of biomechanical properties of cells, tissues, and molecular interaction forces [16]. 

In this framework, AFM and SNOM appear to be valuable tools for the reproductive biologist. Unlike standard or novel imaging techniques, AFM and SNOM are versatile platforms that can acquire multiple pieces of information: topography, high-resolution imaging, and biomechanics. This information allows a better understanding of how morphological, biochemical, and mechanical properties come together to guide cell processes. 

This review collects more recent findings obtained employing these techniques in several conditions affecting human fertility. The potentiality of these microscopies in the analysis of molecular mechanisms by single-molecule manipulation is examined. Finally, the translational relevance of these findings in view of developing diagnostic tools and therapeutic treatments is discussed. 

## 2. AFM and SNOM: The Use of Force and Light

During the past 30 years, scanning probe microscopies (SPM) quickly gained widespread attention, from hard materials science to biology [17]. SPM encompasses a family of surface-sensitive techniques, each of which is based on the interrogation at the nanometer level of a specific physical property by a sharp proximal probe. In tunneling microscopy, this property is the tunneling current between a sharp metallic tip and the conductive sample, while AFM interrogates Van der Waals forces between probe and sample. Further essential details common to SPM are the piezo-elements to control the probe position, the probe-sample separation with the sub-nm resolution, and the feedback system to keep a constant set-point (i.e., current, force, etc.). 

**Figure 1:**
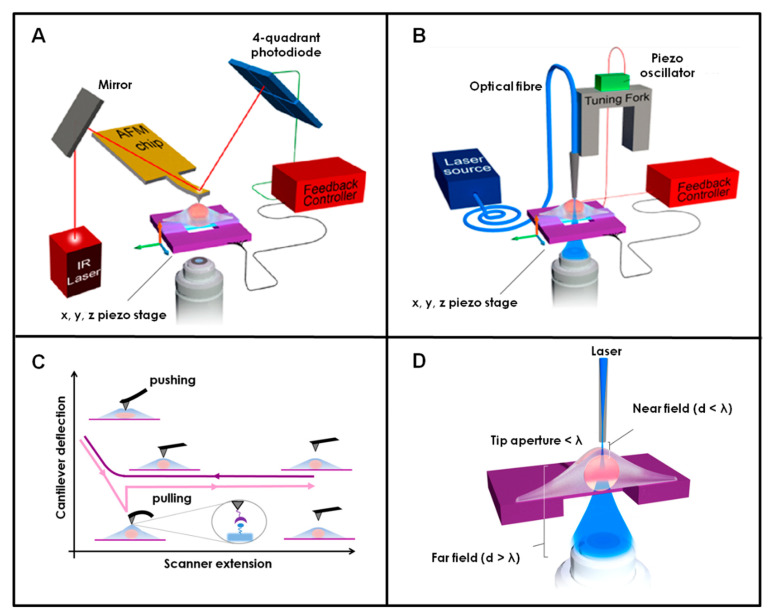
Schematic illustration of the atomic force microscopy (AFM) (**A**) and aperture scanning near-field optical microscopy (SNOM) (**B**) instrumentation with the basic working elements of the two techniques. Force-distance curve (magenta approach and pink retraction trace) showing how the cantilever is brought into contact with the sample, bends and the tip located at the end “pushes” on the sample, thereby enabling one to apply force or indent the sample. When the cantilever is retracted, in the “pulling” phase, interaction forces between tip and sample can be measured, up to molecular level (**C**). Zoomed view of the SNOM probe: the end part of the optical fiber close to the sample surface, where the small aperture of the optical fiber and the local light interaction create the near-field used to reach high resolution (**D**).

In AFM, the probes (conical or pyramidal) have a radius of curvature that ranges from a few nanometers for very sharp tips up to 50 nm, and it is located at the end of a flexible cantilever made of silicon or silicon nitride. Forces acting between the probe and the sample surface cause a tiny cantilever bending that is detected with a position-sensitive detector, which measures the deflection of a laser beam focused on the backside of the cantilever (Figure 1A). By multiplying this laser displacement on a photodetector by the cantilever spring constant, the values of the forces acting between the tip and the sample can be measured with pN sensitivity. These forces may have different origins: the extension of a protein is dominated by entropic forces, the indentation of a cell involves the mechanical response of the cytoskeleton, the antigen-antibody separation requires the rupture of several hydron bonds, the pulling of a membrane tether generates viscous forces, etc. The orders of magnitude of interest are, for instance, 10 pN for covalent bond rupturing, 100 pN for protein unfolding, and 1 nN for cytoskeleton remodeling [18].

In imaging mode, the tip is scanned across the sample surface to obtain a topographical reconstruction. AFM Imaging differs mainly in how the tip moves over the cell sample: contact, intermittent, or non-contact modes: the last two are together referred to also as dynamic modes. In contact mode, the tip is in constant repulsive interaction with the sample surface. This can lead to friction effects, which may drag single proteins or locally modify or damage the cell surface. In dynamic mode, the cantilever is driven at its resonant frequency while the oscillation amplitude and phase are monitored, thereby minimizing the tip-sample interactions. In particular, in non-contact mode the tip vibrates close to the sample surface always in the attractive regime of forces and never touches the surface; in intermittent contact mode the tip oscillates between repulsive and attractive forces, tapping the sample while scanning, but still with reduced friction effects while contouring the biological sample. All these imaging modes can provide nanometric spatial resolution images of cells or single proteins in fluid conditions [19]. The development of high-speed atomic force microscopy (HS-AFM), rapid imaging, and real-time visualization offered the advantage of analyzing the dynamic behavior of biological macromolecules [20]. So far, the current performance of HS-AFM reported is to record movies of molecular motion with up to 15 Hz frame rate, without disturbing the biomolecular functions. 

One of the most relevant advantages of AFM with respect to other microscopic techniques resides in the possibility of sensing and applying a wide range of forces (from pN up to a few μN) corresponding to the ones that dominate biological phenomena, from the single protein to the single cell [21]. In force spectroscopy mode, cantilever bending is recorded while the tip and the sample are approached and retracted (Figure 1C). During the approach, the cantilever will bend upwards when pushed onto the sample, thereby providing force-indentation curves. The fitting of these curves with the Hertz model enables to evaluate the mechanical properties of single cells (e.g., cell stiffness) or the biomechanical features of a tissue [14]. Upon retraction, attractive forces between the tip and the sample can be measured, and various AFM modes can be identified, including single-molecule force spectroscopy (SMFS), molecular recognition mapping (MRM), and single-cell force spectroscopy (SCFS). These spectroscopy modes can be used to quantify and localize interaction forces (e.g., adhesion forces; binding/unbinding forces) of biological systems over scales ranging from cells to single molecules [22] and make AFM a powerful multifunctional tool to study biological processes at the molecular scale [23]. In addition, AFM can be integrated with optical or confocal fluorescence microscopy to correlate mechanical features with cytoplasmic organelles, such as actin cortex organization [24]. 

SNOM combines AFM-like topographical information with super-resolution optical images of the sample. SNOM can operate in both aperture and apertureless modes [25]. In aperture mode, the probe is generally a tiny aperture (20–100 nm in diameter) at the end of a metal-coated tapered optical fiber (Figure 1B) or a hollow AFM cantilever with a subwavelength aperture at its apex, coupled with a guided of free space light source [26]. The optical fiber is brought close to the sample surface at a distance of a fraction of the used wavelength, causing near-field evanescent waves, emitted from the aperture, to be scattered into the far-field, where they are detected (Figure 1D). The intensity of the evanescent wave decays exponentially, moving away from the aperture. As a result, the near-field is localized in a volume within ~100 nm nanometers from the probe. When the tip is brought closer to the sample at a distance less than the decay length of the evanescent field, the sample interacts with the incoming light by absorbing it or scattering it into the far-field, both forward and backward (usually indicated as transmission and reflection, respectively). To a first approximation: transmitted light provides information as dark field optical microscopy does in the far-field, while reflected light provides information similarly to phase imaging, both with better spatial resolution. In this way, SNOM can be exploited to acquire super-resolution optical images by scanning a probe tip over a surface [27]. A peculiar SNOM application is fluorescence. Absorption can be used to produce images in fluorescence, with a surface sensitivity equivalent to that of total internal reflection fluorescence (TIRF) microscopy but better lateral resolution [28]. The potentialities of this technique applied to imaging of fluorescent-labeled single biomolecules are underlined by several papers [29,30], which show the ability of SNOM to map the molecular organization of protein clusters or lipid rafts in the cell membrane, to study their involvement in cell function. Besides, SNOM has been proved to be helpful to describe the interplay between cell function and morphology, such as morphological changes of endothelial cells in inflammatory reaction or particle uptake in epithelial cells [31,32]. 

Apertureless SNOM, on the other hand, is based on light scattering from the tip, and its intensity depends on the tip-sample interaction. Without the need for a fiber aperture and relying on scattered, rather than transmitted light, the tip size can be downscaled to a few nanometers with improvements in spatial resolution [25]. Since the vertical extent of the near field is determined by the tip size, apertureless SNOM cannot be used to investigate the inner structure of a cell. Instead, it finds better applications in the investigation of lipid membranes, cell membranes, single protein, isolated fluorophores, fibrils, DNA, and other nanosized structures [32,33]. 

## 3. Morphological Analysis of Gametes by AFM Imaging

The application of electron microscopy has allowed describing in detail the ultrastructural anomalies of the human spermatozoa (e.g., abnormal head, neck, midpiece, and tail defects) [34,35] and of the human oocytes (e.g., changes of the cytoplasm and zona pellucida), which can affect cell function [36]. In recent years AFM has provided the embryologists with the possibility of obtaining a real 3D highly resolved reconstruction of cells in nearly physiological conditions, without metallization as required for scanning electron microscopy. This achievement has attracted much attention for the analysis of gamete morphology, particularly in the case of spermatozoa. Indeed, interesting results have been obtained by the detailed topographical characterization of specific sperm portions (i.e., acrosome, midpiece, and axoneme) achieved by AFM imaging [37,38,39]. For instance, an abnormal sperm head was associated with improperly packed chromatin, and the analysis of AFM images of human spermatozoa has revealed that, in pathological forms, surface roughness and nuclear thickness are higher than the normal spermatozoa [39]. The evaluation of sperm morphology is a controversial aspect in semen analysis for the determination of a male’s fertility potential. Recent studies have highlighted that sperm morphology has a weak impact on male fertility and low prediction in assisted reproduction practices [40]. Nevertheless, the morphological analysis is still relevant to correlated cellular anomalies, which affect sperm motility and function, with a molecular mechanism, as in spermatozoa, where multiple morphological abnormalities of the flagella are dictated by specific gene mutations [41]. 

High-resolution analysis of surface topography by amplitude (peak–valley height difference, arithmetic roughness, root mean square roughness) and spatial roughness has been demonstrated to help quantify morphological alterations of the spermatozoa and describe the effects of new male contraceptives [42]. A similar high-resolution analysis can be relevant also in some pathology conditions like varicocele. A detailed description of the sperm morphology before and after surgical intervention can be helpful to elucidate the debated molecular mechanisms at the base of this pathological condition, which frequently leads to male infertility [43].

In the oocytes AFM imaging is used to characterize the topography of zona pellucida (ZP). The ZP is a porous network of glycoproteins with a thickness of 10–15 μm that surrounds mammalian oocytes. This peculiar layer has several functions: it regulates the sperm interaction and binding, prevents polyspermy after fertilization, and protects early embryos moving in the female reproductive tract. The penetration of the ZP matrix by spermatozoa is a crucial step in oocyte fertilization, and their inability to penetrate it inevitably leads to infertility. ZP dysmorphology, like dark ZP, large perivitelline space, or irregular-shaped ZP, is associated with reduced implantation and pregnancy rates following IVF compared to normal oocytes [44,45,46]. AFM imaging provides some interesting information about ZP roughness variations in immature, mature, and fertilized oocytes [47]. However, in this case, the imaging has several limitations. To scan the ZP surface, it is necessary to remove the ZP from the oocyte and deposit it onto a support. All these procedures can interfere with glycoprotein organization and compromise the original ZP superficial structure. 

## 4. The Importance of Mechanics in Gametes and Embryos 

### 4.1. The Mechanical Properties of the Oocyte

The mechanics of oocytes deserve particular attention not only for their relevant role in the maturation stages of the oocyte and in the fertilization process, but also for the appealing opportunity to use mechanical features in the screening and selection of oocytes in the assisted reproduction practices. Several papers have reported that the cell’s mechanical phenotype is correlated with its physiological or pathological status [48,49,50]. Therefore, these characteristics have attracted much interest in the design and development of novel diagnostic equipment based on the detection of cell mechanical phenotype for clinical applications [51].

In ARTs, trained embryologists select oocytes to fertilize or the embryo to transfer, mainly by visual inspection via bright field optical microscopy, subjecting the selection to personal interpretation. To improve this selection method, several non-invasive methodologies based on optical imaging techniques have been proposed [6,52,53]. Some of them have considered morphometric and morphokinetic parameters, but their relevance is still debated [52,53]. Another one, based on the detection of the mitotic spindle by polarized light microscopy, shows that visualization of the mitotic spindle can improve the prediction of oocyte quality. However, even in this case, conflicting findings are reported [6,54]. Recently, artificial intelligence (AI)-based methods are emerging as a potential approach for selecting competent oocytes and the best embryo to transfer during IVF [55].

The mechanical properties of the oocytes could represent non-invasive, quantitative, and objective parameters that might help embryologists with selecting the most competent oocyte for fertilization.

Mammalian oocytes go through a maturation process to develop the competencies needed to be fertilized. Oogenesis occurs during the fetal period, resulting in the formation of oocytes arrested in prophase I of meiosis I. Later, after hormonal stimulation (i.e., ovulation), the maturation proceeds to form secondary oocytes (MII stage) that can be fertilized by a sperm cell. The ZP surrounds the oocytes in all of these maturation stages, and it changes its mechanical properties after fertilization, with a process called “zonal hardening.” This phenomenon was confirmed by mechanical measurements on whole oocytes [56] and isolated ZP layers by indentation measurements performed by AFM [57]. Further measurements revealed both a stiffening of the ZP and the occurrence of the elastic-to-plastic transition at larger forces [47]. These mechanical changes are associated with the molecular arrangements of the glycoprotein components of the ZP. They are described in the biological process of fertilization but not well clarified in some pathological conditions as a postovulatory aging of the oocyte [58]. The postovulatory oocyte’s aging has a notable impact on fertilization potential, and although several factors are known to be involved in its regulation, the exact molecular mechanisms that control these processes remain elusive [58,59,60]. In this sense, the quantification of the oocyte mechanical properties could aid in correlating the mechanical properties’ variations with the molecular events inside the oocyte in similar conditions.

Different approaches are used to investigate the mechanical properties of whole oocytes, such as micropipette indentation and aspiration [56,60,61,62,63,64], which indicate that oocyte stiffness can vary not only after fertilization but even at different maturation stages and with aging [56,64]. Although these techniques provide important information about oocytes’ mechanical properties, they have some limitations mostly related to the high deformation applied during measurements, making the oocyte’s different structural elements (i.e., ZP, perivitelline space, and ooplasm) indistinguishable from the mechanical point of view.

**Figure 2:**
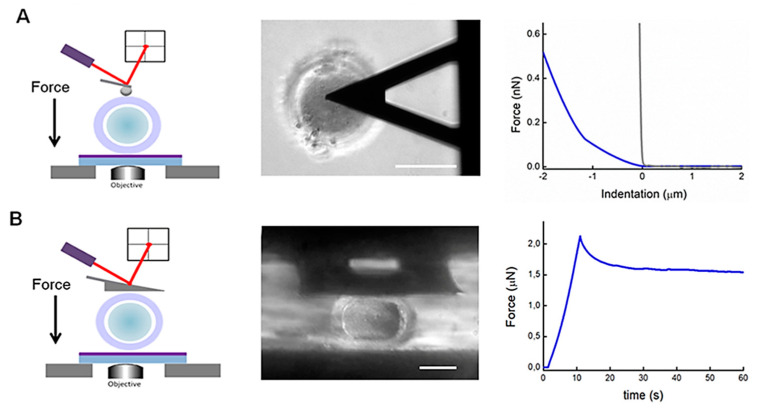
AFM indentation measurements with a micrometer bead on an oocyte: a scheme, a bright field image (top view), and a representative force-indentation curve obtained on a human oocyte (blue line) and a rigid surface (gray line) displayed for comparison (**A**). AFM stress-relaxation measurements with a macrocantilever (square 300 × 300 μm^2^) on an oocyte: a scheme, a bright field image (side-view), and a representative stress-relaxation curve obtained from a human oocyte (**B**). Scale bar 50 μm.

For that reason, AFM appears as an ideal tool, since it allows fine control of both the applied forces (pN-nN range) and the probe displacement (nm range). Moreover, as already highlighted for somatic cells [15,65], probes with different sizes and shapes can help identify the mechanical properties of the distinct structural elements of the oocyte [66]. The AFM probes with a sharp conical or pyramidal shape (common tip radius 10–25 nm) are not well suited for the oocyte. A probe with such a small contact area would likely penetrate the meshed glycoprotein structure of the ZP external layer. More suitable probes to study oocyte mechanics are a micrometer-sized glass beads attached to tipless cantilevers (Figure 2A), or even better, macro-cantilevers with large flat areas at the cantilever end (300 × 300 μm^2^ in size), as shown in Figure 2B, that provide larger contact areas. AFM indentation measurements with a bead of 4.5 μm in diameter frequently present double-trended force-indentation curves likely associated with two different mechanical responses (Figure 2A). For this reason, the curves are fitted with a modified double Hertz model [67]. As a result, the first section of the curve provides a Young’s modulus (E1) likely associated with the compression of the outer superficial part of ZP, while the second one (E2) corresponds to the rest of the ZP oocyte. The elastic response of the ZP outer layer is different and distinguishable from the rest of ZP-HO for all maturation stages. Furthermore, these elasticity values are observed to change with maturation stage and the quality of the oocytes [67]. Similar AFM indentation measurements on vitrified oocytes confirmed this mechanical response and demonstrate that the elasticity of cryopreserved oocyte remains unchanged after thawing and is not affected by the freezing procedure [68].

The E values obtained by AFM indentation on the whole oocyte are lower than those obtained with other techniques (see Table 1). This trend is also observed when evaluating the stiffness of somatic cells, through which relevant variations of E values are found using different techniques and theoretical models [15]. The distributed E values indicate that the mechanical response of the oocyte depends on the force applied and the extent of deformation. For a large deformation, the mechanical response can involve more structural elements, while with bead indentation, the response is mainly determined by the ZP. Moreover, it is essential to consider that the cell viscoelasticity affects the resulting E values, as observed when measurements are carried out at different loading rates [15].

Macro-cantilevers have enabled some to investigate and evaluate the viscoelastic properties of the whole oocyte (Figure 2B). Stress-relaxation measurements were performed on human and mouse oocytes by lowering the z-piezo 20 μm beyond the contact point. In both cases, the analysis of the force-relaxation curves showed two distinct relaxation processes: a fast (1–2 s) and a slower relaxation (10–20 s) [69]. The presence of the two relaxation times suggests that the two main structural components of the oocyte, the ZP and the ooplasm, contribute differently to the mechanical response of the oocyte. A similar trend was also observed by Shen and co-authors [66], who proposed that by applying a reduced model, the mechanical contribution of the ZP can be distinguished from the ooplasm by performing consecutive measurements with different sizes of indenter: a small indenter to determine the ZP mechanical parameters, and a large one to provide the ooplasm’s mechanical contribution [66].

In the mechanical analysis of the oocyte, some authors draw attention to another peculiar structure: the cumulus cell–oocyte complex matrix [70]. This structure is an extended coat that forms around the oocyte a few hours before ovulation and contains the hyaluronic matrix with embedded cumulus cells. The analysis of the mechanical properties of this coat performed by indentation-based AFM reveals peculiar mechanical characteristics of this area. In vivo and in vitro extreme softness (0.55 ± 0.1 Pa and 1.65 ± 0.3 Pa) were evaluated, together with a tendency to stiffen under compressive stress. They also observed that these features were associated with a peculiar ultrastructure: the cumulus cell–oocyte complex appeared to be surrounded by a thick matrix shell that was essentially devoid of cumulus cells. The authors stated the relevance of the heterogeneity and mechanics of this area during transport through the oviduct for the correct selection, capture, and guidance of sperm.

### 4.2. Embryo: Mechanotransduction and Mechanical Properties

During embryonic development, mechanotransduction phenomena are crucial, as are chemical signals, to control morphogenesis and tissue patterning [71]. Investigations about lumenal pressure changes, blastocyst cavity volume variation, and stiffness of the trophoblast have demonstrated how mechanical cues guide the early stages of embryo development and blastocyst expansion [72,73,74]. 

The physical features of the microenvironment regulate differentiation and self-renewal of pluripotent stem cells [75], and cell–cell contact (i.e., adhesion points, actin-myosin contractility) is crucial to dictate cell coordination and tissue organization [76]. It is not entirely clear what the magnitudes and distribution of forces are within embryos and how they trigger mechanosensitive responses by cells that guide embryo development. Advancing techniques to perform precise measurement and manipulation of these forces could be essential to address this question and understanding the molecular mechanisms that trigger and drive cell differentiation. 

In this context, AFM can be used to investigate the effects of forces in embryo development. The inner pressure and the E values of different blastocyst regions are measured using laser-assisted magnetic tweezers in combination with AFM. During the progression of a mouse blastocyst, from embryonic day E3.0 (16 cells) to E4.5 (>100 cells), the inner pressure of the embryo increases from 100 to 500 Pa; E values of the trophoblast from 300 to 600 Pa; and from 50 to 250 Pa for the inner cell mass. All of these variations are related to the actin organization [77]. 

More recently, Thompson et al. performed time-lapse in vivo atomic force microscopy (tiv-AFM) to obtain time-resolved measurements of developmental tissue mechanics. Tiv-AFM combines sensitive upright epi-fluorescence imaging of opaque samples and iterated AFM indentations of in vivo tissue at a time resolution of tens of minutes [78]. On this timescale they observed significant changes in tissue stiffness, related to important implications for specific biological processes, such as the outgrowth of axons by retinal ganglion cells across the developing embryonic brain.

Like for oocytes, there are significant efforts to improve embryo selection, and many of the optical and AI approaches proposed for oocytes are also considered valid for this choice [52,53,55]. The mechanical properties of embryos can represent a parameter helpful for identifying viable, high-quality embryos to implant. Yanez and co-workers have demonstrated that embryo potential is strictly associated with the quality of the fertilized oocyte, and the embryo’s mechanical properties themselves represent predictive factors of zygote life and birth success in the mouse [64]. For this reason, biomechanical properties were proposed as a promising new approach to have a quantitative and objective estimate of embryo developmental potential [60,79].

## 5. The Mechanical Properties of the Cells and Tissues in Female Reproductive Disorders

Several pathologies or inflammation of the female reproductive system severely affect the women’s fertility. The investigation of their hallmarks is of relevance to better understand these diseases. Mechanics are a fundamental feature of the female reproductive system. The organs of this system exhibit astonishing mechanical performance, given their remarkable strength, and undergo huge deformations in their physiological activity. It is also well known that mechanical alterations of the uterus and cervix are associated with pathologies and can induce preterm delivery [80]. Advances in the mechanical characterization of these organs’ tissues are crucial. Indeed, they can provide unrevealed information and help develop new detection and treatment strategies for common diseases that affect women’s health, such as uterine cancer, endometriosis, and ovarian cancer.

Ovarian cancer is one of the most frequent gynecological malignancies. In total, 12% of ovarian cancer is diagnosed in fertile women, and an early-stage detection of the pathology is vital to increase the survival rate and allow the patients to find a way to preserve fertility [81]. However, no effective screening methods to detect early-stage disease are present, and it is usually only diagnosed when already widespread in the abdomen. The epithelial cell adhesion molecule (EpCAM) is over-expressed on the surfaces of several cancer cells, including ovary tumors, and has been regarded as a marker for tumor diagnosis and therapy. To this end, it is very interesting to investigate its interactions with aptamers—small DNA molecules that can be used to target these proteins similarly to antibodies, but they are smaller and have higher stability. In this case, SMFS is exploited to investigate the recognition process between the small aptamer (SYL3C) and EpCAMs. As a result, the interaction forces and the binding kinetics of these biomolecules are described [82]. These studies provide further insights into molecular interactions and help in designing novel targeted cancer diagnostic, prognostic, and therapeutic strategies based on EpCAMs.

The biomechanical characterization of non-malignant immortalized ovarian surface epithelium cells (IOSE) and four ovarian cancer cell lines, performed by AFM, demonstrated that cancer cells exhibit a lower mean stiffness than non-malignant ones. Moreover, it was observed that increases in invasiveness and migratory behavior are both correlated with a significant reduction in cell stiffness [83], similarly to what is observed in other cancer types. Consequently, this indicates that the cell stiffness represents an additional effective biomarker for detecting highly aggressive ovarian cancer cells. Moreover, the analysis of epithelial ovarian cancer cells on hydrogel substrates with different rigidity demonstrated that an increased microenvironment mechanical stiffness enhances cell migration, suggesting a relationship between matrix stiffness and disease progression [84]. 

Furthermore, AFM was used to mechanically characterize four human ovarian tissues distinct pathological conditions: mucinous and serous cystadenoma, mature teratoma, and endometriosis [85]. As expected, the authors found considerable heterogeneity within the tissue, but they could observe some differences in elastic modulus values of the cellular part and extracellular matrix among the four ovarian samples. These findings support that mechanical measurements represent a valuable tool to delineate the mechanical phenotypes of cells and tissue in ovary tumors. Based on the biomechanical properties, a screening approach could be employed to complement standard biopsy procedures, offering a novel and quantitative diagnostic approach to help cancer grading/classification, with an unbiased evaluation of the sample [85,86].

A further challenging development of the mechanobiological approach in reproductive medicine research could be the investigation of the mechanical properties of the uterus tissue. The uterus elasticity analysis could shed some light on several issues, from embryo implantation troubles and miscarriage, to the painful endometriosis pathology. Biomechanical investigations of the interested tissues may offer a deeper understanding of the phenomena and provide alternatives for early diagnosis and follow-up treatments.

## 6. New Insights in Reproductive Medicine from the Study of Single-Molecule Interactions

For twenty years, AFM-SMFS experiments, with an exceptionally high spatial and temporal resolution, have been extensively utilized to investigate the intramolecular and intermolecular protein interactions in a large variety of biological processes: folding/unfolding; force-dependent kinetics in molecular interactions; and protein–protein, protein–DNA, and protein–drug interactions [87,88,89]. They have all contributed to clarifying molecular mechanisms at the basis of cellular functions, which are of great interest to medical research.

SMFS remains poorly exploited in the reproductive medicine field, although its application could clarify central issues of the field such as the initial sperm–oocyte interaction, binding, and gamete fusion. Three specific glycoproteins in mice and four in humans are the principal components of the ZP; specifically, ZP2 is involved in sperm binding [90,91]. These studies are carried out mainly via fluorescence microscopy, electron microscopy, and standard molecular biology methodologies. The application of force spectroscopy assay would offer the opportunity to study this process in real-time and on the living cells. These measurements would give quantitative information on the sperm–ZP2 interaction forces and the dynamics of the binding process. Moreover, a precise cell manipulation could clarify the occurrence of possible mechanotransduction events in the exocytosis of cortical granules, a phenomenon associated with oocyte activation [92]. A recent work highlighted how adhesion molecules both on the sperm and oocyte accumulate in an adhesion area and can play a principal role in the gamete fusion—by using a combination of time-lapse confocal microscopy and a pipette assay, with which they observed an enrichment of the adhesion proteins in the contact region [93]. Although they provided essential information, they could not quantify the contact force and adhesion energy involved in the process. Furthermore, it has not been investigated whether mechanical variations of oolemma might play a role in this protein recruitment.

The SMFS application could also be extended to investigate the interactions of the adhesion proteins involved in tumor pathologies of the female reproductive system to achieve new information about the molecular mechanisms that trigger and guide tumor cell migration and metastatic process.

## 7. From Ultrastructure to Single-Molecule Visualization in the Reproductive System

Advancements in the description of biological processes of interest in reproductive medicine can be achieved by integrating topological information with the visualization of the inner cellular organization up to single molecules. 

### 7.1. Inner Cellular Organization of Spermatozoa

**Figure 3:**
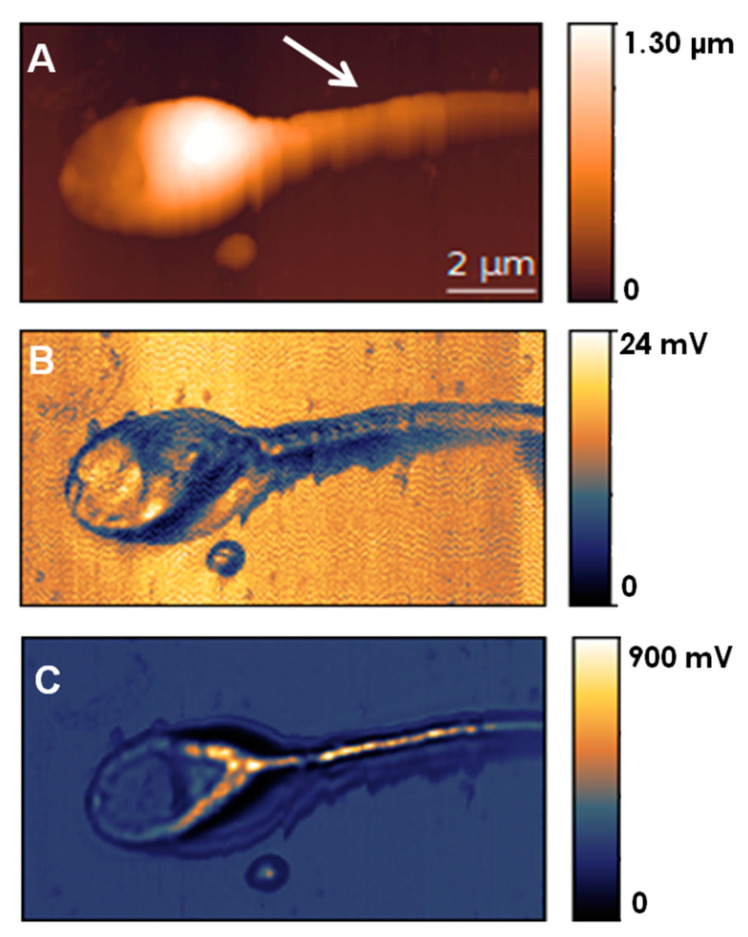
SNOM topography (**A**) reflection (**B**) and transmission (**C**) images of human spermatozoa with normal features, as modified from [95] with permission. The arrow in (**A**) indicates the sperm region in which the mitochondria’s helicoidally arrangement can be discerned in the corresponding SNOM transmission image (**C**).

By using aperture-SNOM, a detailed ultrastructural description of the human spermatozoa was achieved. With this technique peculiar structural features of these cells have been disclosed without staining procedures. As shown in Figure 3, the helicoidal organization of mitochondria in the midpiece region is well discernible in normal spermatozoa [94], and other structural features of healthy and anomalous spermatozoa are detected, all well resembling those observed in transmission electron microscopy images [95]. These works demonstrate the ability of SNOM to visualize inner cellular compartments with a typical contrast (transmission and reflection) for standard microscopy but with a higher resolution [94,95]. The ability to detect differences in the optical contrast of cellular structures and avoid standard staining protocols is very appealing, as staining can mask structural features in some cases at very high resolutions. Although this effect is limited in the analysis of cellular components, a staining fluorophore or fluorophore-antibody can interfere with the actual size and interactions of a single protein investigated. SNOM high resolution might be particularly useful in investigating sperm ultra-structures that still need to be clarified better to understand their relations with sperm function [96].

### 7.2. Diseases of the Female Reproductive System

Besides the high optical resolution, SNOM opens the opportunity to access nano-spectrochemical information of single proteins, which would broaden our knowledge about the relation between their structure and function [97]. In this context, the SNOM application may aid in diagnosing several pathologies related to the female reproductive system. 

In the cervical tumor, the histopathology classification is made using four discriminatory features: (1) the ratio of nuclei to the cytoplasm, (2) the diameter of nuclei, (3) the shape factor, and (4) the compactness of nuclei. Once these features are identified in the cervical biopsy images, they are classified into normal, pre-cancer (cervical intraepithelial neoplasia—CIN1, CIN2, and CIN3), and malignant. However, the diagnosis has some flaws related to subjective visual inspection, as with other tumor screenings. Recently, Halliwell et al. reported how the combination of scanning near-field optical microscopy with an infrared free-electron laser (SNOM-IR-FEL) aided in distinguishing between normal and squamous low-grade and high-grade dyskaryosis, and between normal and mixed squamous/glandular pre-invasive and cervical adenocarcinoma lesions, by considering specific wavelengths associated with DNA, amide I/II, and lipids [98]. Their findings highlighted how the SNOM-IR-FEL technique might help obtain relevant chemical information to detect cervical cell abnormalities and perform cancer diagnosis at a high spatial resolution [99].

### 7.3. Diseases of the Male Reproductive System and Fertility

**Figure 4:**
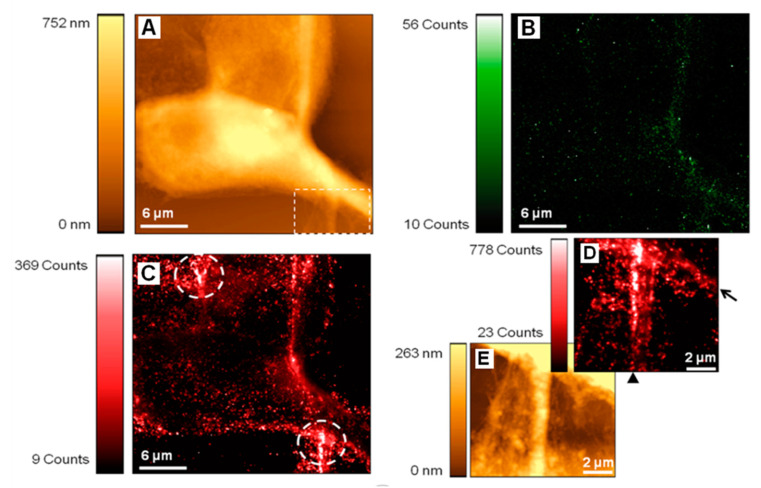
SNOM topography (**A**,**E**) and fluorescence (**B**–**D**) images of PNT2 epithelial cells with dual-labeling, which, thanks to the use of two separated filters, enables one to identify the location of the tight junction protein ZO-1 (in green) and E-cadherin (in red). The region included in the box in (**A**) was scanned at a higher resolution to generate the detailed E-cadherin fluorescence image (**D**) with the corresponding topography in (**E**). Circled regions in (**C**) and the arrow/arrowhead in (**D**) highlight E-cadherin clusters. The picture was published with permission [100].

SNOM has been demonstrated to be helpful in visualizing and localizing adhesion molecules in prostate cancer cells. Understanding how individual constituents of cell surfaces localize and assemble is crucial, since the spatial organization and interactions of the cell surface machinery tightly control their functions in physiological and pathological conditions. In this sense, the application of SNOM in combination with innovative labeling strategies such as quantum dots enables the molecular localization of membrane proteins at a high resolution of epithelial prostate cancer cells. Specifically, SNOM acquisitions with a double-labeling allow protein co-localization on the cell surface, as shown in Figure 4 [100]. Thanks to this approach, the cellular localization of the cell–cell adhesion proteins E-cadherins in healthy prostate epithelial cells and cancer cells was precisely described. The different molecular distributions of E-cadherins between healthy epithelial prostate cells and cancer cells coincide with markedly different morphologies and the absence of cell–cell interactions of cancer cells with the neighboring cells [100]. 

A further important issue in the assessment of male and female infertility is the analysis of chromosomal aberrations. In this framework, SNOM covers the resolution gap between far-field light and electron microscopy on the supra-molecular morphological analysis of meiotic chromosomes. SNOM combined with immunofluorescent staining enables the visualization of the morphology of the telomere end of meiotic chromosomes and identifies structural and functional proteins located in this region [101]. Telomeres, repeated sequences at the ends of chromosomes, widely investigated for their involvement in genome instability and aging, are attracting growing interest for the possible role of their structure and length in spermatogenesis [102]. High-resolution analysis of these chromosomal regions provides information on telomere length and proteins associated with these sequences. These features can be considered as biomarkers that correlate with male fertility potential.

Although SNOM bears appealing perspective in reproductive medicine and biology, its application is still poor due to some limitations. Optimizing staining protocols is essential in some cases. Besides, the high resolution is difficult to maintain in the liquid when scanning in the aperture mode configuration. Owing to tip-sample interactions, the apex aperture can easily widen, resulting in a super optical resolution loss. Furthermore, in shear-force mode and other contact operations in general, SNOM requires long scan times for high-resolution imaging. However, the instrument is constantly evolving to achieve the best configuration to work in biology. Recent advances in the technique propose a combination of high-speed AFM and SNOM. By attaching gold nanoparticles to a home-made tip built on a standard AFM cantilever and a total internal reflection system, the authors achieved an imaging rate of ~3 s/frame for SNOM, more than 100-times higher than the typical SNOM imaging rate. The same group also demonstrated a ~39 nm resolution in high-speed SNOM imaging of fluorescently labeled DNA in solution [103]. 

## 8. The Impact of AFM and SNOM Findings on Reproductive Medicine

Given the growing number of couples turning to reproductive medicine and the not fully satisfying IVF success rate, there is a need to improve assisted reproductive methods. One of the major issues encountered in IVF is selecting the competent oocyte and embryo to achieve a positive outcome. Many studies focused on identifying biomarkers that can improve IVF outcomes are currently ongoing, but objective, accurate, and noninvasive assessments are still missing in clinical practice. Moreover, there is still 25% idiopathic infertility, whose causes are unknown. Likely, they are associated with abnormalities or altered molecular mechanisms which are not detected or described by current methods. All these issues demand new methodological analysis and advanced techniques to solve and overcome these problems. 

In the basic research of reproductive medicine, AFM and SNOM instrumentation can help clarify molecular mechanisms. However, they cannot be directly applied in clinical practices: they still are time-consuming, expensive, not automatized, and require well-trained operators, but the findings obtained by these techniques are of great relevance to improving our knowledge about gametes and disorders related to reproductive organs. Like optical and spectroscopic features, the mechanical parameters can be considered additional and might be exploited in diagnostic assays and ARTs. Furthermore, due to the enormous potential of such microscopies, numerous efforts are devoted to developing automated systems, such as AFM, which can perform the acquisition of numerous force-distance curves to measure the biomechanical properties of large cell populations or tissues [104]. 

The combination of biological analysis with biophysical methodologies in the future will contribute to improving our knowledge about molecular mechanisms and open up new possibilities for screening, diagnosis, surgical, and medical treatments in reproductive medicine.

## Figures and Tables

**Table 1:** Methodologies used to evaluate the Young’s modulus of the oocyte.

Method	Force Loading/Pressure Applied	Deformation/Aspiration Length/ Indentation	Young Modulus	Model
Micropipette indentation				
	7.5 µN	44 µm	17.9 kPa	Biomembrane Point Load model [61]
	0.5 μN	35 µm	3.1 kPa	Maugis-Dugdale model [62]
Micropipette aspiration				
	0.8 kPa	40 µm	2.41 kPa	Cortical shell-liquid core model [63]
	2.20 kPa	16 µm	0.056 N/m	Zener model [64]
Microtactile sensor (MTS)				
		10 µm	8.26 kPa	Hertz model [56]
AFM-indentation				
	0.001–0.002 μN	1–2 µm	0.05–0.1 kPa	Double Hertz model [67,68]

## References

[1] Smith G.D., Takayama S. (2017). Application of microfluidic technologies to human assisted reproduction. Mol. Hum. Reprod..

[2] Pascolo L., Bedolla D.E., Vaccari L., Venturin I., Cammisuli F., Gianoncelli A., Mitri E., Giolo E., Luppi S., Martinelli M. (2016). Pitfalls and promises in FTIR spectromicroscopy analyses to monitor iron-mediated DNA damage in sperm. Reprod. Toxicol..

[3] Pascolo L., Zupin L., Gianoncelli A., Giolo E., Luppi S., Martinelli M., De Rocco D., Sala S., Crovella S., Ricci G. (2019). XRF analyses reveal that capacitation procedures produce changes in magnesium and copper levels in human sperm. Nucl. Instrum. Methods Phys. Res. Sect. B Beam Interact. Mater. Atoms.

[4] Zupin L., Pascolo L., Gianoncelli A., Gariani G., Luppi S., Giolo E., Ottaviani G., Crovella S. (2020). Ricci Synchrotron radiation soft X-ray microscopy and low energy X-ray fluorescence to reveal elemental changes in spermatozoa treated with photobiomod-ulation therapy. Anal. Methods.

[5] Pachetti M., Zupin L., Venturin I., Mitri E., Boscolo R., D’Amico F., Vaccari L., Crovella S., Ricci G., Pascolo L. (2020). FTIR Spectroscopy to Reveal Lipid and Protein Changes Induced on Sperm by Capacitation: Bases for an Improvement of Sample Selection in ART. Int. J. Mol. Sci..

[6] Jasensky J., Swain J.E. (2013). Peering Beneath the Surface: Novel Imaging Techniques to Noninvasively Select Gametes and Em-bryos for ART. Biol. Reprod..

[7] Ajduk A., Szkulmowski M. (2019). Light microscopy of mammalian gametes and embryos: Methods and applications. Int. J. Dev. Biol..

[8] D’Amico F., Zucchiatti P., Latella K., Pachetti M., Gessini A., Masciovecchio C., Vaccari L., Pascolo L. (2020). Investigation of genomic DNA methylation by ultraviolet resonant Raman spectroscopy. J. Biophotonics.

[9] Mallidis C., Sanchez V., Wistuba J., Wuebbeling F., Burger M., Fallnich C., Schlatt S. (2014). Raman microspectroscopy: Shining a new light on reproductive medicine. Hum. Reprod..

[10] Schermelleh L., Ferrand A., Huser T., Eggeling C., Sauer M., Biehlmaier O., Drummen G.P.C. (2019). Super-resolution micros-copy demystified. Nat. Cell Biol..

[11] Sherlekar A., Mundhe G., Richa P., Dey B., Sharma S., Rikhy R. (2020). F-BAR domain protein Syndapin regulates actomyosin dynamics during apical cap remodeling in syncytial Drosophila embryos. J. Cell Sci..

[12] Lim C.T., Bershadsky A., Sheetz M.P. (2010). Mechanobiology. J. R. Soc. Interface.

[13] Moeendarbary E., Harris A.R. (2014). Cell mechanics: Principles, practices, and prospects. Wiley Interdiscip. Rev. Syst. Biol. Med..

[14] Schwarz U.S. (2017). Mechanobiology by the numbers: A close relationship between biology and physics. Nat. Rev. Mol. Cell Biol..

[15] Wu P.-H., Aroush D.R.-B., Asnacios A., Chen W.-C., Dokukin M.E., Doss B.L., Durand-Smet P., Ekpenyong A., Guck J., Guz N.V. (2018). A comparison of methods to assess cell mechanical properties. Nat. Methods.

[16] Krieg M., Fläschner G., Alsteens D., Gaub B.M., Roos W.H., Wuite G.J.L., Gaub H.E., Gerber C., Dufrêne Y.F., Müller D.J. (2019). Atomic force microscopy-based mechanobiology. Nat. Rev. Phys..

[17] Raigoza A.F., Dugger J.W., Webb L.J. (2013). Recent Advances and Current Challenges in Scanning Probe Microscopy of Bio-molecular Surfaces and Interfaces. Appl. Mater. Interfaces.

[18] Roca-Cusachs P., Conte V., Trepat X. (2017). Quantifying forces in cell biology. Nat. Cell Biol..

[19] Dufrêne Y.F., Ando T., Garcia R., Alsteens Y.F.D.D., Martinez-Martin D., Engel A., Gerber C., Müller D.M.-M.D.J. (2017). Imaging modes of atomic force microscopy for application in molecular and cell biology. Nat. Nanotechnol..

[20] Ando T. (2017). Directly watching biomolecules in action by high-speed atomic force microscopy. Biophys. Rev..

[21] Alsteens D., Gaub H.E., Newton R., Pfreundschuh M., Gerber C., Müller D.J. (2017). Atomic force microscopy-based characteri-zation and design of biointerfaces. Nat. Rev. Mat..

[22] Müller D.J., Dufrêne Y.F. (2011). Atomic force microscopy: A nanoscopic window on the cell surface. Trends Cell Biol..

[23] Dufrêne Y.F., Pelling A.E. (2013). Force nanoscopy of cell mechanics and cell adhesion. Nanoscale.

[24] Rheinlaender J., Dimitracopoulos A., Wallmeyer B., Kronenberg N.M., Chalut K.J., Gather M.C., Betz T., Charras G., Franze K. (2020). Cortical cell stiffness is independent of substrate mechanics. Nat. Mater..

[25] Bazylewski P., Ezugwu S., Fanchini G. (2017). A Review of Three-Dimensional Scanning Near-Field Optical Microscopy (3D-SNOM) and Its Applications in Nanoscale Light Management. Appl. Sci..

[26] Biagioni P., Celebrano M., Polli D., Labardi M., Zavelani-Rossi M., Cerullo G., Finazzi M., Duò L. (2007). Nonlinear optics and spectroscopy at the nanoscale with a hollow-pyramid aperture SNOM. J. Phys. Conf. Ser..

[27] Richards D. (2003). Near-field microscopy: Throwing light on the nanoworld. Philos. Trans. R. Soc. A Math. Phys. Eng. Sci..

[28] Nagy P., Jenei A., Kirsch A.K., Szöllosi J., Damjanovich S., Jovin T.M. (1999). Activation-dependent clustering of the erbB2 recep-tor tyrosine kinase detected by scanning near-field optical microscopy. J. Cell Sci..

[29] Hinterdorfer P., Garcia-Parajo M.F., Dufrêne Y.F. (2012). Single-Molecule Imaging of Cell Surfaces Using Near-Field Nanoscopy. Accounts Chem. Res..

[30] De Lange F., Cambi A., Huijbens R., De Bakker B., Rensen W., Garcia-Parajo M., Van Hulst N., Figdor C.G. (2001). Cell biology beyond the diffraction limit: Near-field scanning optical. J. Cell Sci..

[31] Bulat K., Rygula A., Szafraniec E., Ozaki Y., Baranska M. (2016). Live endothelial cells imaged by Scanning Near-field Optical Microscopy (SNOM): Capabilities and challenges. J. Biophotonics.

[32] Zhang Y., Kyle J.R., Penchev M., Yazdanpanah V., Yu J., Li Y., Yang M., Budak G., Ozbay E., Ozkan M. (2012). Transmission Near-Field Scanning Optical Microscopy Investigation on Cellular Uptake Behavior of Iron Oxide Nanoparticles. BioNanoScience.

[33] Naumenko D., Cassese D., Lazzarino M., Bek A., Benfenati F. (2014). Novel Approaches for Single Molecule Activation and Detection. Advances in Atom and Single Molecules Machine.

[34] Mortimer D. (2018). The functional anatomy of the human spermatozoon: Relating ultrastructure and function. Mol. Hum. Reprod..

[35] Ricci G., Andolfi L., Zabucchi G., Luppi S., Boscolo R., Martinelli M., Zweyer M., Trevisan E. (2015). Ultrastructural Morphology of Sperm from Human Globozoospermia. BioMed Res. Int..

[36] Nottola S.A., Macchiarelli G., Familiari G. (2014). Fine Structural Markers of Human Oocyte Quality in Assisted Reproduction. Austin J. Reprod. Med. Infertil..

[37] Kumar S., Chaudhury K., Sen P., Guha S.K. (2005). Atomic force microscopy: A powerful tool for high-resolution imaging of spermatozoa. J. Nanobiotechnol..

[38] Mai A., Weerachatyanuku W., Tomietto M., Wayner D.D.M., Wells G., Balhorn R., Leader A., Cyr J.L., Tanphaichitr N. (2002). Use of Atomic Force Microscopy for Morphological and Morphometric Analyses of Acrosome Intact and Acrosome-Reacted Human Sperm. Mol. Reprod. Dev..

[39] Sunanda P., Panda B., Dash C., Padhy R.N., Routray P. (2018). An illustration of human sperm morphology and their functional ability among different group of subfertile males. Andrology.

[40] Danis R.B., Samplaski M.K. (2019). Sperm Morphology: History, Challenges, and Impact on Natural and Assisted Fertility. Curr. Urol. Rep..

[41] Tang S., Wang X., Li L., Yang X., Li Z., Liu W., Li C., Zhu Z., Wang L., Wang J. (2017). Biallelic Mutations in CFAP43 and CFAP44 Cause Male Infertility with Multiple Morphological Abnormalities of the Sperm Flagella. Am. J. Hum. Genet..

[42] Kumar S., Chaudhury K., Sen P., Guha S.K. (2007). Quantitative analysis of surface micro-roughness alterations in human sper-matozoa using atomic force microscopy. J. Microsc..

[43] Jensen C.F.S., Østergren P., Dupree J.M., Ohl D.A., Sønksen J., Fode M. (2017). Varicocele and male infertility. Nat. Rev..

[44] Gabrielsen A., Lindenberg S., Petersen K. (2001). The impact of zona pellucida thickness variation of human embryos on preg-nancy outcome in relation to suboptimal embryo development. A prospective randomized controlled study. Hum. Reprod..

[45] Bianchi V., Macchiarelli G., Borini A., Lappi M., Cecconi S., Miglietta S., Familiari G., Nottola S.A. (2014). Fine morphological assessment of quality of human mature oocytes after slow freezing or vitrification with a closed device: A comparative analysis. Reprod. Biol. Endocrinol..

[46] Sauerbrun-Cutler M.-T., Vega M., Bręborowicz A., Gonzales E., Stein D., Lederman M., Keltz M. (2015). Oocyte zona pellucida dysmorphology is associated with diminished in-vitro fertilization success. J. Ovarian Res..

[47] Papi M., Brunelli R., Sylla L., Parasassi T., Monaci M., Maulucci G., Missori M., Arcovito G., Ursini F., De Spirito M. (2009). Mechanical properties of zona pellucida hardening. Eur. Biophys. J..

[48] Cross S.E., Jin Y.-S., Rao J., Gimzewski J.K. (2007). Nanomechanical analysis of cells from cancer patients. Nat. Nanotechnol..

[49] Andolfi L., Bourkoula E., Migliorini E., Palma A., Pucer A., Skrap M., Scoles G., Beltrami A.P., Cesselli D., Lazzarino M. (2014). Investigation of Adhesion and Mechanical Properties of Human Glioma Cells by Single Cell Force Spectroscopy and Atomic Force Microscopy. PLoS ONE.

[50] Lekka M. (2016). Discrimination Between Normal and Cancerous Cells Using AFM. BioNanoScience.

[51] Rosendahl P., Plak K., Jacobi A., Kraeter M., Toepfner N., Otto O., Herold C., Winzi M., Herbig M., Ge Y. (2018). Real-time fluorescence and deformability cytometry. Nat. Methods.

[52] Faramarzi A., Khalili M.A., Omidi M. (2017). Morphometric analysis of human oocytes using time lapse: Does it predict embryo developmental outcomes?. Hum. Fertil..

[53] Barberet J., Bruno C., Valot E., Antunes-Nunes C., Jonval L., Chammas J., Choux C., Ginod P., Sagot P., Soudry-Faure A. (2019). Can novel early non-invasive biomarkers of embryo quality be identified with time-lapse imaging to predict live birth?. Hum. Reprod..

[54] Caamano J.N., Munoz M., Diez C., Gomez E. (2010). Polarized Light Microscopy in Mammalian Oocytes. Reprod. Dom. Anim..

[55] Zaninovic N., Rosenwaks Z. (2020). Artificial intelligence in human in vitro fertilization and embryology. Fertil. Steril..

[56] Murayama Y., Mizuno J., Kamakura H., Fueta Y., Nakamura H., Akaishi K., Anzai K., Watanabe A., Inui H., Omata S. (2006). Mouse zona pellucida dynamically changes its elasticity during oocyte maturation, fertilization and early embryo development. Hum. Cell.

[57] Boccaccio A., Frassanito M.C., Lamberti L., Brunelli R., Maulucci G., Monaci M., Papi M., Pappalettere C., Parasassi T., Sylla L. (2012). Nanoscale characterization of the biomechanical hardening of Bovine zona pellucida. J. R. Soc. Interface.

[58] Miao Y.-L., Kikuchi K., Su Q.-Y., Schatten H. (2009). Oocyte aging: Cellular and molecular changes, developmental potential and reversal possibility. Hum. Reprod..

[59] Lord T., Aitken R.J. (2013). Oxidative stress and ageing of the post-ovulatory oocyte in Reproduction. Reproduction.

[60] Yanez L.Z., Camarillo D.B. (2017). Microfluidic analysis of oocyte and embryo biomechanical properties to improve outcomes in assisted reproductive technologies. Mol. Hum. Reprod..

[61] Sun Y., Wan K.-T., Roberts K.P., Bischof J.C., Nelson B.J. (2003). Mechanical property characterization of mouse zona pellucida. IEEE Trans. NanoBiosci..

[62] Liu X., Shi J., Zong Z., Wan K.-T., Sun Y. (2012). Elastic and Viscoelastic Characterization of Mouse Oocytes Using Micropipette Indentation. Ann. Biomed. Eng..

[63] Khalilian M., Navidbakhsh M., Valojerdi M.R., Chizari M., Yazdi P.E. (2009). Estimating Young’s modulus of zona pellucida by micropipette aspiration in combination with theoretical models of ovum. J. R. Soc. Interface.

[64] Yanez L.Z., Han J., Behr B.B., Reijo Pera R.A., Camarillo D.B. (2016). Human oocyte developmental potential is predicted by mechanical properties within hours after fertilization. Nat. Commun..

[65] Krause M., Te Riet J., Wolf K. (2013). Probing the compressibility of tumor cell nuclei by combined atomic force–confocal microscopy. Phys. Biol..

[66] Shen T., Benet E., Sridhar S.L., Abadie J., Piat E., Vernerey F.J. (2019). Separating the contributions of zona pellucida and cyto-plasm in the viscoelastic response of human oocytes. Acta Biomater..

[67] Andolfi L., Masiero E., Giolo E., Martinelli M., Luppi S., Zilio S.D., Delfino I., Bortul R., Zweyer M., Ricci G. (2016). Investigating the mechanical properties of zona pellucida of whole human oocytes by atomic force spectroscopy. Integr. Biol..

[68] Giolo E., Martinelli M., Luppi S., Romano F., Ricci G., Lazzarino M., Andolfi L. (2019). Study of the mechanical properties of fresh and cryopreserved individual human oocytes. Eur. Biophys. J..

[69] Andolfi L., Greco S.L.M., Tierno D., Chignola R., Martinelli M., Giolo E., Luppi S., Delfino I., Zanetti M., Battistella A. (2019). A novel AFM macro-probe to study the biomechanical properties of large cells and 3D cell spheroids. Acta Biomater..

[70] Chen X., Bonfiglio R., Banerji S., Jackson D.G., Salustri A., Richter R.P. (2016). Micromechanical Analysis of the Hyaluronan-Rich Matrix Surrounding the Oocyte Reveals a Uniquely Soft and Elastic Composition. Biophys. J..

[71] Mammoto T., Ingber D.E. (2010). Mechanical control of tissue and organ development. Development.

[72] Piccolo S. (2013). Mechanics in the embryo. Nat. Cell Biol..

[73] Maître J.-L. (2017). Mechanics of blastocyst morphogenesis. Biol. Cell.

[74] Chan C.J., Costanzo M., Ruiz-Herrero T., Mönke G., Petrie R.J., Bergert M., Diz-Muñoz A., Mahadevan L., Hiiragi T. (2019). Hydraulic control of mammalian embryo size and cell fate. Nat. Cell Biol..

[75] D’Angelo M., Benedetti E., Tupone M.G., Catanesi M., Castelli V., Antonosante A., Cimini A. (2019). The Role of Stiness in Cell Reprogramming: A Potential Role for Biomaterials in Inducing Tissue Regeneration. Cells.

[76] Heisenberg C.-P., Bellaïche Y. (2013). Forces in Tissue Morphogenesis and Patterning. Cell.

[77] Wang X., Zhang Z., Tao H., Liu J., Hopyan S., Sun Y. (2018). Characterizing Inner Pressure and Stiffness of Trophoblast and Inner Cell Mass of Blastocysts. Biophys. J..

[78] Thompson A.J., Pillai E.K., Dimov I.B., Foster S.K., Holt C.E., Franze K. (2019). Rapid changes in tissue mechanics regulate cell behaviour in the developing embryonic brain. eLife.

[79] Kort J., Behr B. (2017). Biomechanics and developmental potential of oocytes and embryos. Fertil. Steril..

[80] Baah-Dwomoh A., McGuire J., Tan T., De Vita R. (2016). Mechanical Properties of Female Reproductive Organs and Supporting Connective Tissues: A Review of the Current State of Knowledge. Appl. Mech. Rev..

[81] Kim S.-Y., Lee J.R. (2016). Fertility preservation option in young women with ovarian cancer. Future Oncol..

[82] Wang N., Liu H., Hao J., Bai X., Li H., Zhang Z., Wang H., Tang J. (2015). Single molecular recognition force spectroscopy study of a DNA aptamer with the target epithelial cell adhesion molecule. Analyst.

[83] Xu W., Mezencev R., Kim B., Wang L., McDonald J., Sulchek T. (2012). Cell Stiffness Is a Biomarker of the Metastatic Potential of Ovarian Cancer Cells. PLoS ONE.

[84] McKenzie A.J., Hicks S.R., Svec K.V., Naughton H., Edmunds Z.L., Howe A.K. (2018). The mechanical microenvironment regulates ovarian cancer cell morphology, migration, and spheroid disaggregation. Sci. Rep..

[85] Ansardamavandi A., Tafazzoli- Shadpour M., Omidvar R., Nili F. (2020). An AFM-Based Nanomechanical Study of Ovarian Tis-sues with Pathological Conditions. Int. J. Nanomed..

[86] Stylianou A., Lekka M., Stylianopoulos T. (2018). AFM assessing of nanomechanical fingerprints for cancer early diagnosis and classification: From single cell to tissue level. Nanoscale.

[87] Wang C., Hu R., Morrissey J.J., Kharasch E.D., Singamaneni S. (2017). Single Molecule Force Spectroscopy to Compare Natural versus Artificial Antibody-Antigen Interaction. Small.

[88] Koehler M., Fis A., Gruber H.J., Hinterdorfer P. (2019). AFM-Based Force Spectroscopy Guided by Recognition Imaging: A New Mode for Mapping and Studying Interaction Sites at Low Lateral Density. Methods Protoc..

[89] Yang B., Liu Z., Liu H., Nash M.A. (2020). Next Generation Methods for Single-Molecule Force Spectroscopy on Polyproteins and Receptor-Ligand Complexes. Front. Mol. Biosci..

[90] Avella M.A., Baibakov B., Dean J. (2014). A single domain of the ZP2 zona pellucida protein mediates gamete recognition in mice and humans. J. Cell Biol..

[91] Tokuhiro K., Dean J. (2018). Glycan-Independent Gamete Recognition Triggers Egg Zinc Sparks and ZP2 Cleavage to Prevent Polyspermy. Dev. Cell.

[92] Baibakov B., Gauthier L., Talbot P., Rankin T.L., Dean J. (2007). Sperm binding to the zona pellucida is not sufficient to induce acrosome exocytosis. Development.

[93] Chalbi M., Barraud-Lange V., Ravaux B., Howan K., Rodriguez N., Soule P., Ndzoudi A., Boucheix C., Rubinstein E., Wolf J.P. (2014). Binding of sperm protein Izumo1 and its egg receptor Juno drives Cd9 accumulation in the intercellular contact area prior to fusion during mammalian fertilization. Development.

[94] Andolfi L., Trevisan E., Troian B., Prato S., Boscolo R., Giolo E., Luppi S., Martinelli M., Ricci G., Zweyer M. (2015). The application of scanning near field optical imaging to the study of human sperm morphology. J. Nanobiotech..

[95] Troian B., Boscolo R., Ricci G., Lazzarino M., Zito G., Prato S., Andolfi L. (2020). Ultra-structural analysis of human spermatozoa by aperture scanning near-field optical microscopy. J. Biophotonics.

[96] Fishman E.L., Jo K., Nguyen Q.P.H., Kong D., Royfman R., Cekic A.R., Khanal S., Miller A.L., Simerly C., Schatten G. (2018). A novel atypical sperm centriole is functional during human fertilization. Nat. Commun..

[97] Yong Y.-C., Wang Y.-Z., Zhong J.-J. (2018). Nano-spectroscopic imaging of proteins with near-field scanning optical microscopy (NSOM). Curr. Opin. Biotechnol..

[98] Halliwell D.E., Morais C.L.M., Lima K.M.G., Trevisan J., Siggel-King M.R.F., Craig T., Ingham J., Martin D.S., Heys K.A., Kyrgiou M. (2016). Imaging cervical cytology with scanning near-field optical microscopy (SNOM) coupled with an IR-FEL. Sci. Rep..

[99] Craig T., Smith A.D., Holder G.M., Ingham J., Smith C.I., Varro A., Pritchard D.M., Barrett S.D., Martin D.S., Harrison P. (2018). SNOM Imaging of a Crypt-Like Feature in Adenocarcinoma Associated with Barrett’s Oesophagus. Phys. Status Solidi B.

[100] Walker K.-A.D., Morgan C., Doak S.H., Dunstan P.R. (2012). Quantum Dots for Multiplexed Detection and Characterisation of Prostate Cancer Cells Using a Scanning Near-Field Optical Microscope. PLoS ONE.

[101] Hausmann M., Liebe B., Perner B., Jerratsch M., Greulich K.-O., Scherthan H. (2003). Imaging of human meiotic chromosomes by scanning near-field optical microscopy (SNOM). Micron.

[102] Rocca M.S., Foresta C., Ferlin A. (2018). Telomere length: Lights and shadows on their role in human reproduction. Biol. Reprod..

[103] Umakoshi T., Fukuda S., Iino R., Uchihashi T., Ando T. (2020). High-speed near-field fluorescence microscopy combined with high-speed atomic force microscopy for biological studies. Biochim. Biophys. Acta (BBA) Gen. Subj..

[104] Coronado S.P., Severac C., Martinez-Rivas A., Dague E. (2019). Beyond the paradigm of nanomechanical measurements on cells using AFM: An automated methodology to rapidly analyse thousands of cells. Nanoscale Horiz..

